# Supplementary material for: Impaired spatial memory in adult vitamin D deficient BALB/c mice is associated with reductions in spine density, nitric oxide, and neural nitric oxide synthase in the hippocampus
Source: AIMS Neurosci. 2022 Jan 26;9(1):31–56. doi: 10.3934/Neuroscience.2022004 (PMC8941191; doi:10.3934/Neuroscience.2022004)
Supplement: Supplementary file 1 [file neurosci-09-01-004-s001.pdf]

Neuroscience

AIMS Neuroscience, 9 (1): 31–56.
DOI: 10.3934/Neuroscience.2022004
Received: 24 November 2021
Revised: 11 January 2022
Accepted: 20 January 2022
Published: 26 January 2022

http://www.aimspress.com/journal/neuroscience

Research article

Impaired spatial memory in adult vitamin D deficient BALB/c mice is associated with reductions in spine density, nitric oxide, and neural nitric oxide synthase in the hippocampus

Md. Mamun Al-Amin[1], Robert K. P. Sullivan[1], Suzy Alexander[1,2], David A. Carter[1], DanaKai Bradford[1,3] and Thomas H. J. Burne[1, 2,]*

[1] Queensland Brain Institute, The University of Queensland, Brisbane 4072, Australia
[2] Queensland Centre for Mental Health Research, Wacol 4076, Australia
[3] Australian E-Health Research Centre, CSIRO, Pullenvale 4069, Australia

* **Correspondence:** Email: t.burne@uq.edu.au; Tel: +61 733466371; Fax: +61 733466301.

Supplementary information

Supplementary Table S1 Composition of Speciality feed Diet (SF09-088 AIN93G Rodent Diet)

Supplementary Table S1.1. Calculated Nutritional Parameters.

Nutrient	Amount
Protein	19.40%
Total Fat	7.00%
Crude Fibre	4.70%
AD Fibre	4.70%
Digestible Energy	16.1 MJ/Kg
% Total calculated digestible energy from lipids	15.90%
% Total calculated digestible energy from protein	21.10%

Supplementary Table S1.2. Base components.

Name of the Ingredients	Rate of inclusion
Casein (Acid)	200 g/Kg
Sucrose	100 g/Kg
Soya Bean Oil	70 g/Kg
Cellulose	50 g/Kg
Maize Starch	404 g/Kg
Dextrinised Starch	132 g/Kg
DL Methionine	3.0 g/Kg
Calcium Carbonate	13.1 g/Kg
Sodium Chloride	2.6 g/Kg
AIN93 Trace Minerals	1.4 g/Kg
Potassium Citrate	2.5 g/Kg
Potassium Dihydrogen Phosphate	6.9 g/Kg
Potassium Sulphate	1.6 g/Kg
Choline Chloride (75%)	4.1 g/Kg
Oxicap E2	0.14 g/Kg
AIN93 Vitamins	15 g/Kg
Vitamin K 0.23%	0.87 g/Kg

Supplementary Table S1.3. Calculated total vitamins.

Name of the Vitamin	Rate of inclusion
Vitamin A (Retinol)	6000 IU/Kg
Vitamin D (Cholecalciferol)#	None added (Deficient)<br>1500 IU/Kg (Control)
Vitamin E (a Tocopherol acetate)	115 mg/Kg
Vitamin K (Menadione)	3.5 mg/Kg
Vitamin C (Ascorbic acid)	None added
Vitamin B1 (Thiamine)	9.1 mg/Kg
Vitamin B2 (Riboflavin)	9.3 mg/Kg
Niacin (Nicotinic acid)	45 mg/Kg
Vitamin B6 (Pyridoxine)	11 mg/Kg
Pantothenic acid	24.5 mg/Kg
Biotin	300 µg/Kg
Folic acid	3 mg/Kg
Vitamin B12 (Cyanocobalamin)	152 µg/Kg
Choline	2380 mg/Kg

#Vitamin D was not given to the Adult vitamin D deficient mice for 10 weeks. However, control mice received 1500 IU/Kg for the same duration.

Supplementary Table S1.4. Calculated Amino Acids.

Name of the Amino acids	Rate of inclusion
Valine	1.26%
Leucine	1.80%
Isoleucine	0.87%
Threonine	0.79%
Methionine	0.84%
Cystine	0.05%
Lysine	1.49%
Phenylalanine	0.99%
Tyrosine	1.01%
Tryptophan	0.27%
Histidine	0.60%

Supplementary Table S1.5. Calculated Total Minerals.

Name of the Minerals	Rate of inclusion
Calcium	0.47%
Phosphorus	0.35%
Magnesium	0.08%
Sodium	0.15%
Chloride	0.16%
Potassium	0.40%
Sulphur	0.23%
Iron	68 mg/Kg
Copper	7.0 mg/Kg
Iodine	0.2 mg/Kg
Manganese	19 mg/Kg
Zinc	46 mg/Kg
Molybdenum	0.15 mg/Kg
Selenium	0.3 mg/Kg
Chromium	1.0 mg /Kg
Fluoride	1.0 mg/Kg
Lithium	0.1 mg/Kg
Boron	2.5 mg/Kg
Nickel	0.5 mg/Kg
Vanadium	0.1 mg/Kg

Supplementary Table S1.6. Calculated Fatty Acid Composition.

**Name of the Minerals**	**Rate of inclusion**
Myristic Acid 14:0	Trace
Palmitic Acid 16:0	0.72%
Stearic Acid 18:0	0.27%
Palmitoleic Acid 16:1	0.01%
Oleic Acid 18:	1.60%
Gadoleic Acid 20:1	0.01%
Linoleic Acid 18:2 n6	3.57%
a Linolenic Acid 18:3 n3	0.48%
Total n3	0.48%
Total n6	3.57%
Total Mono Unsaturated Fats	1.62%
Total Polyunsaturated Fats	4.05%
Total Saturated Fats	0.99%

AIMS Press © 2022 the Author(s), licensee AIMS Press. This is an open access article distributed under the terms of the Creative Commons Attribution License (http://creativecommons.org/licenses/by/4.0)
